# Simultaneous peroral endoscopic myotomy and submucosal tunneling endoscopic septum division in one tunnel for diffuse esophageal spasm combined with epiphrenic diverticulum

**DOI:** 10.1055/a-2638-3229

**Published:** 2025-07-15

**Authors:** Shao-Bin Luo, Zu-Qiang Liu, Li Wang, Quan-Lin Li, Ping-Hong Zhou

**Affiliations:** 1 92323 Endoscopy Center and Endoscopy Research Institute, Zhongshan Hospital, Fudan University, Shanghai, China; 2 92323 Shanghai Collaborative Innovation Center of Endoscopy, Shanghai, China; 3 Endoscopy Center, Shanghai Geriatric Medical Center, Shanghai, China

A 78-year-old female patient was admitted with dysphagia for 2 years. High-resolution esophageal manometry confirmed diffuse esophageal spasm (DES). Endoscopy showed multiple narrow rings in the middle and lower esophagus lumen, tight stenosis at the esophagogastric junction (EGJ), and a epiphrenic diverticulum (ED) located above the narrowed EGJ ( Fig. 1 **a–c** ). Given the DES accompanied by ED, peroral endoscopic myotomy (POEM) combined with submucosal tunneling endoscopic septum division (STESD) was performed ( Video 1 ). After establishing the submucosal tunnel, the annular muscle bundle and diverticular ridge were completely transected ( Fig. 1 **d–g** ), and the whole layer of esophageal muscle bundle was completely severed 2 cm above and below the EGJ ( Fig. 1 **h–k** ). Substantial reduction of lower esophageal sphincter tonus was confirmed by easy passage of the EGJ through the endoscope. The length of the tunnel and muscle incision is 18 and 15 cm, respectively. The patient was discharged on postoperative day 3 without complication. One year after the operation, a follow-up endoscopy confirmed the disappearance of the diverticulum and narrow rings, with a smooth passage of the EGJ ( Fig. 1 **l** ).

**Fig. 1:**
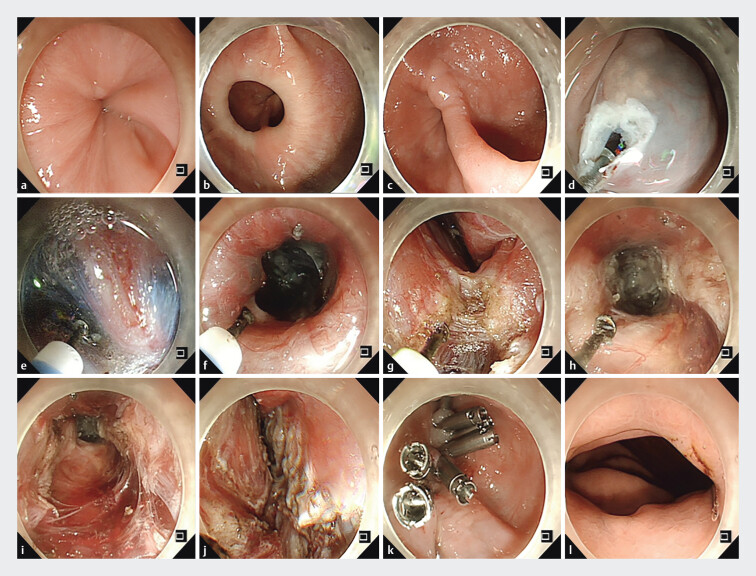
**a** Tightly closed gastroesophageal junction. **b** Narrow rings of diffuse esophageal spasm. **c** An epiphrenic diverticulum. **d, e** Creation of a submucosal tunnel. **f, g** Completion of the full-thickness myotomy. **h–j** The muscle of the diverticulum septum was completely cut off. **k** The tunnel entrance was closed by metal clips. **l** One year after operation, the disappearance of the large diverticulum and narrow rings, with a smooth passage of the EGJ.

Simultaneous POEM and STESD in one tunnel for diffuse esophageal spasm combined with epiphrenic diverticulum.Video 1

This case is the first report about simultaneous POEM and STESD in one tunnel for DES with ED. In this case, POEM may not be enough to resolve the symptoms of dysphagia, necessitating combined STESD for ED. Moreover, incision of diverticular ridge and spastic muscle layer in the same tunnel should avoid mucosal injury and esophageal perforation, which greatly increases the difficulty of operation. ED is currently thought to be secondary to an underlying esophageal motility disorder (EMD), such as DES or achalasia 1 . The traditional treatment for EMD combined with ED is laparoscopic epiphrenic diverticulectomy, myotomy, and fundoplication, with high postoperative morbidity and mortality 2 . This implies that the application of simultaneous POEM and STESD in one tunnel may be a safe and effective technique for DES combined with ED.

Endoscopy_UCTN_Code_TTT_1AO_2AG_3AZ

## Notes

Endoscopy E-Videos https://eref.thieme.de/e-videos
*E-Videos* is an open access online section of the journal *Endoscopy* , reporting on interesting cases and new techniques in gastroenterological endoscopy. All papers include a high-quality video and are published with a Creative Commons CC-BY license. Endoscopy E-Videos qualify for HINARI discounts and waivers and eligibility is automatically checked during the submission process. We grant 100% waivers to articles whose corresponding authors are based in Group A countries and 50% waivers to those who are based in Group B countries as classified by Research4Life (see: https://www.research4life.org/access/eligibility/ ). This section has its own submission website at https://mc.manuscriptcentral.com/e-videos .
